# The PrpF protein of *Shewanella oneidensis* MR-1 catalyzes the isomerization of 2-methyl-*cis*-aconitate during the catabolism of propionate via the AcnD-dependent 2-methylcitric acid cycle

**DOI:** 10.1371/journal.pone.0188130

**Published:** 2017-11-16

**Authors:** Christopher J. Rocco, Karl M. Wetterhorn, Graeme S. Garvey, Ivan Rayment, Jorge C. Escalante-Semerena

**Affiliations:** 1 Department of Bacteriology, University of Wisconsin–Madison, Madison, WI, United States of America; 2 Department of Biochemistry, University of Wisconsin–Madison, Madison, WI, United States of America; 3 Department of Microbiology, University of Georgia, Athens, GA, United States of America; Universidade Nova de Lisboa Instituto de Tecnologia Quimica e Biologica, PORTUGAL

## Abstract

The 2-methylcitric acid cycle (2-MCC) is a common route of propionate catabolism in microorganisms. In *Salmonella enterica*, the *prpBCDE* operon encodes most of the 2-MCC enzymes. In other organisms, *e*.*g*., *Shewanella oneidensis* MR-1, two genes, *acnD* and *prpF* replace *prpD*, which encodes 2-methylcitrate dehydratase. We showed that together, *S*. *oneidensis* AcnD and PrpF (*So*AcnD, *So*PrpF) compensated for the absence of PrpD in a *S*. *enterica prpD* strain. We also showed that *So*AcnD had 2-methylcitrate dehydratase activity and that PrpF has aconitate isomerase activity. Here we report *in vitro* evidence that the product of the *So*AcnD reaction is an isomer of 2-methyl-*cis*-aconitate (2-MCA], the product of the *Se*PrpD reaction. We show that the *So*PrpF protein isomerizes the product of the AcnD reaction into the PrpD product (2-MCA], a known substrate of the housekeeping aconitase (AcnB]. Given that *So*PrpF is an isomerase, that *So*AcnD is a dehydratase, and the results from *in vivo* and *in vitro* experiments reported here, it is likely that 4-methylaconitate is the product of the AcnD enzyme. Results from *in vivo* studies using a *S*. *enterica prpD* strain show that *So*PrpF variants with substitutions of residues K73 or C107 failed to support growth with propionate as the sole source of carbon and energy. High-resolution (1.22 Å) three-dimensional crystal structures of PrpF^K73E^ in complex with *trans-*aconitate or malonate provide insights into the mechanism of catalysis of the wild-type protein.

## Introduction

**Fig 1:**
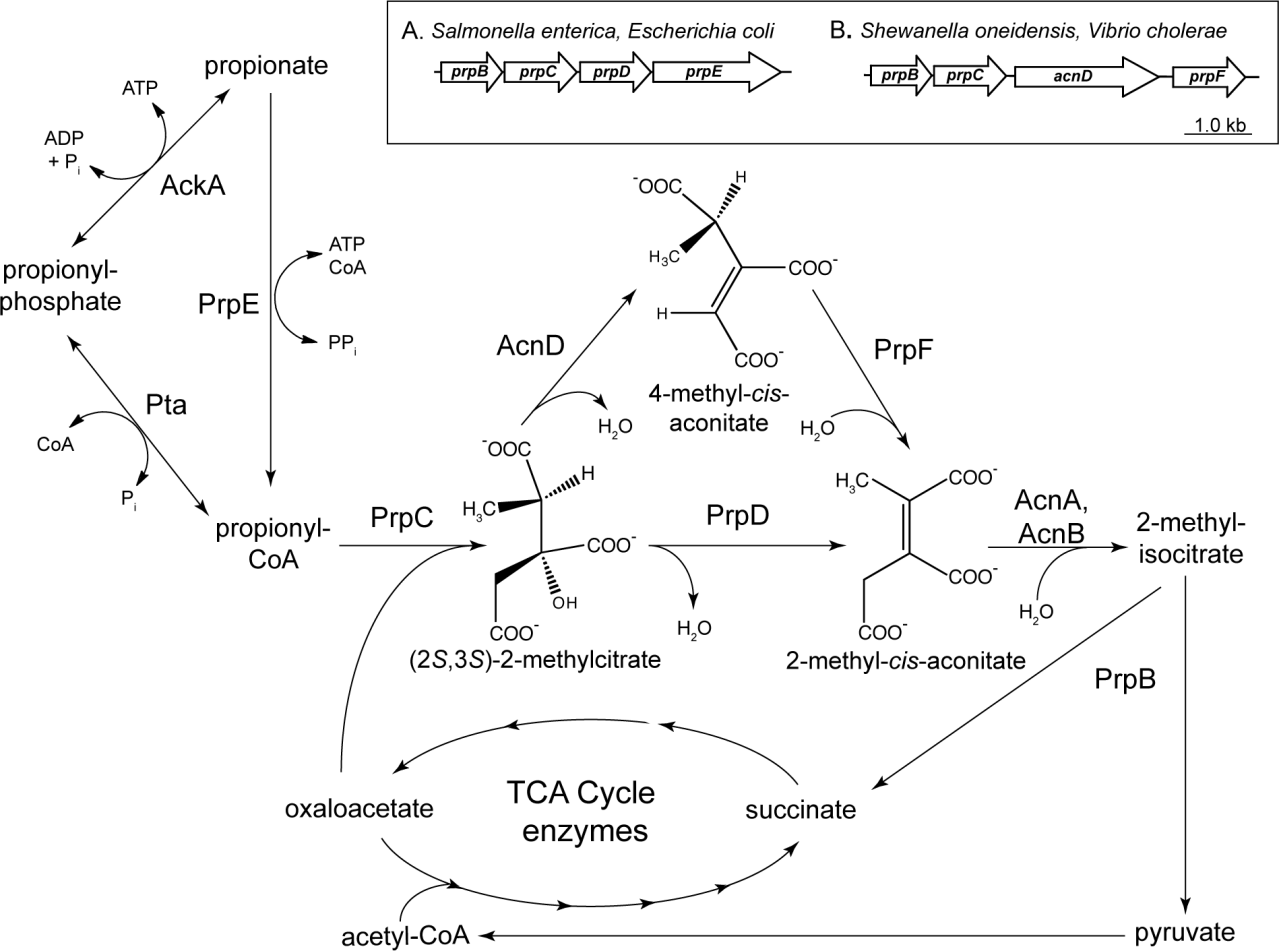
The 2-methylcitric acid cycle. In this metabolic pathway propionate is oxidized to pyruvate with succinate as byproduct. Inset box–difference in operon structure of *Salmonella enterica* and *Escherichia coli* (Inset box A) compared to *Shewanella oneidensis* and *Vibrio cholera* (Inset box B). AckA–acetate kinase; Pta–phosphotransacetylase; AcnA/B–aconitase A, aconitase B.

The 2-methylcitric acid cycle (2-MCC) (Fig 1) is widely distributed route of propionate catabolism in microorganisms. Originally identified in the fungus *Candida lipolytica* (*Yarrowia lipolytica*) [1], the 2-MCC has been characterized in Gamma-proteobacteria (*e*.*g*., *Salmonella enterica*, *Escherichia coli*) [2–4], actinobacteria (*e*.*g*. *Mycobacterium tuberculosis*, *Mycobacterium smegmatis*, *Corynebacterium glutamicum*) [5–9], and Beta-proteobacteria (*e*.*g*. *Ralstonia eutropha*, *Burkholderia sacchari*) [10, 11]. Most of the enzymes that comprise the 2-MCC are encoded as an operon [12]. In *S*. *enterica* (and many other bacteria), the operon consists of four genes, in the order *prpBCDE*. *prpB* encodes 2-methylisocitrate lyase (EC 4.2.1.99) [2, 13, 14]; *prpC* encodes the 2-methylcitrate synthase (EC 2.3.3.5) (2); *prpD*, encodes the 2-methylcitrate dehydratase (EC 4.2.1.79) [15]; and *prpE* encodes the propionyl-CoA synthetase (EC 6.2.1.17) [16, 17].

There is one notable variation in bacterial *prp* operons. That is, some *prp* operons lack the 2-methylcitrate dehydratase *prpD*, but rather contain two other genes, *acnD* and *prpF* [15]. AcnD has aconitase-like activity [18], whilst PrpF has aconitate isomerase activity [19] (Fig 1). In *S*. *oneidensis* the sequence of *prp* genes is *prpR prpB prpC acnD prpF* compared to the *S*. *enterica* sequence *prpR prpB prpC prpD prpE*. In *S*. *oneidensis*, the *prpE* gene encoding propionyl-CoA synthetase is >1.5 Mbp away from the *prp* operon. AcnD and PrpF activities are necessary and sufficient to compensate for the lack of PrpD during growth with propionate of a *prpD* strain of *S*. *enterica* [18].

Structural and biochemical analyses of *Shewanella oneidensis* PrpF (hereafter *So*PrpF), revealed a role of *So*PrpF in the isomerization of *trans-*aconitate to *cis-*aconitate [19], leading us to propose that the role of *So*PrpF in the 2-MCC was to change the stereochemistry of the *S*. *oneidensis* AcnD (hereafter *So*AcnD) reaction product. That is, *So*PrpF was proposed to have 2-methylaconitate isomerase activity [19].

Herein we specifically address the hypothesis that the *S*. *oneidensis* PrpF (*So*PrpF) protein isomerizes the product of the *So*AcnD dehydratase (putatively 4-methyl-*cis-*aconitate (4-MCA) to 2-methyl-*cis-*aconitate (2-MCA), the substrate of aconitase A, B. To facilitate this work, we used a *S*. *enterica* Δ*prpD* carrying the wild-type allele of the *S*. *oneidensis acnD* gene. Into the *S*. *enterica* Δ*prpD /* p*acnD*^+^ (*S*. *oneidensis*) strain we introduced a second plasmid encoding wild-type or variants of *S*. *oneidensis* PrpF (*So*PrpF). In this heterologous system, PrpF functionality was assessed *in vivo* under conditions that demanded propionate utilization as the sole source of carbon and energy, or a combination of propionate and succinate, which would allow us to assess the effect of the accumulation of 4-MCA on growth in the presence of functional or dysfunctional PrpF proteins.

## Materials and methods

### Chemicals and bacteria culture media

All chemicals were purchased from Sigma Chemical Co. unless otherwise stated. 2-Methylcitrate was purchased from CDN Isotopes (Pointe-Claire, Canada). Authentic 2-methyl-*cis*-aconitate was custom synthesized by AsisChem (Cambridge, MA). The experiments reported here were performed in well-characterized *S*. *enterica prp* mutant strains. A previous report from our laboratory showed that this approach allowed us to assess *S*. *oneidensis* AcnD and PrpF functions *in vivo* [18]. *E*. *coli* strains used to overproduce recombinant proteins were grown in lysogeny broth (LB) [20, 21]. No-carbon essential (NCE) medium [22, 23] was used as minimal medium, and was supplemented with MgSO_4_ (1 mM) and methionine (0.5 mM). When added to rich medium, antibiotic concentrations were: ampicillin (100 μg/ml), kanamycin (50 μg/ml), and chloramphenicol (25 μg/ml). Bacto^TM^ Agar (Difco, 1.5% w/v) was used as medium solidifying agent.

For cloning purposes, restriction endonuclease NdeI was purchased from Fermentas (Glen Burnie, MD), and BamHI was purchased from Promega (Madison, WI). Cloning was performed in *E*. *coli* strain DH5α/F’ (New England Biolabs). Plasmids were introduced into *S*. *enterica* strains by electroporation. Cultures were grown in LB medium to an optical density (650 nm) ~0.6–0.8, 1.0 ml of culture was centrifuged at 18,000 x *g* using a Microfuge 18 Centrifuge (Beckman Coulter), cells were washed three times with 1.0 ml of ice-cold sterile water, and re-suspended in 100 μl of water. Plasmids were electroporated into cells using a Bio-Rad Gene Pulser (Hercules, CA) according to the manufacturer’s recommendations. Strains and plasmids used in this study are listed in Table 1.

**Table 1:** Strains and plasmids used in this study.

Strain	Relevant genotype	Source
***E*. *coli* strains**		
BL21(λDE3)	F^-^ *ompT hsdS**b* (r_b_^-^ m_b_^+^) *dcm gal* l(DE3)	New England Biolabs
DH5α/F’	F’/*endA1 hsdR17* (r_k_^-^ m_k_^+^) *supE44 thi-1 recA1 gyrA* (Nal^r^) *relA1* Δ(*lacZYA-argF*)*U169 deoR* [f80d*lac*D(*lacZ*)*M15*]	New England Biolabs
XL10-Gold® Ultracompetent cells	TetR Δ(*mcrA*)183Δ(*mcrCB-hsdSMR-mrr*)173 *endA1 supE44 thi-1 recA1 gyrA96 relA1 lac* Hte [F' *proAB lacIqZ*Δ*M15* Tn*10* (TetR) Amy CamR]a	Stratagene
		
***S*. *enterica* strains**		
TR6583	*metE205 ara-9*	K. Sanderson via J. Roth
		
**Derivatives of TR6583**		
JE8250	*prpD126*::*cat*^*+*^	This work
JE8256	JE8250 / pPRP138	This work
JE8429	JE8256 / pPRP153	This work
JE9373	JE8256 / pPRP215 *prpF255*	This work
JE9374	JE8256 / pPRP216 *prpF256*	This work
JE9592	JE8256 / pPRP218 *prpF257*	This work
JE9593	JE8256 / pPRP219 *prpF258*	This work
JE11597	JE8256 / pPRP223 *prpF259*	This work
		
**Plasmids**		
pPRP138	*S*. *oneidensis acnD*^+^ in pBAD30 *bla*^+^	[18]
pPRP153	*S*. *oneidensis prpF*^+^ in pBAD18Kan *kan*^+^	[18]
pPRP195	*S*. *oneidensis acnD*^*+*^ cloned into pET-15b *bla*^*+*^	This work
pPRP196	*S*. *oneidensis prpF*^*+*^ cloned into pTEV4 *bla*^*+*^	[19]
pPRP205	*S*. *oneidensis acnD*^*+*^ cloned into pTEV5	This work
pPRP215	*S*. *oneidensis prpF*^+^(*prpF255* C107A) in pBAD18Kan *kan*^+^	This work
pPRP216	*S*. *oneidensis prpF*^+^(*prpF256*; encodes PrpF^C107S^) cloned into pBAD18-Kan *kan*^+^	This work
pPRP217	*S*. *oneidensis prpF*^+^(*prpF255*; encodes PrpF^C107A^) cloned into pTEV4 *bla*^*+*^	This work
pPRP218	*S*. *oneidensis prpF*^+^(*prpF257*; encodes PrpF^K73A^) cloned into pBAD18-Kan *kan*^+^	This work
pPRP219	*S*. *oneidensis prpF*^+^(*prpF258*; encodes PrpF^K73E^) cloned into pBAD18-Kan *kan*^+^	This work
pPRP220	*S*. *oneidensis prpF*^+^(*prpF257*; encodes PrpF^K73A^) cloned into pTEV4 *bla*^*+*^	This work
pPRP221	*S*. *oneidensis prpF*^+^(*prpF258*; encodes PrpF^K73E^) cloned into pTEV4 *bla*^*+*^	This work
pPRP223	*S*. *oneidensis prpF*^+^(*prpF259*; encodes PrpF^K73M^) cloned into pBAD18-Kan *kan*^+^	This work
pET-15b	Cloning vector pBR origin of replication *bla*^*+*^; fuses a thrombin-cleavable H_6_ tag to the N-terminus of the protein of interest	Novagen
pTEV4	Cloning vector; F1 origin of replication *bla*^*+*^; fuses a TEV-cleavable H_6_ tag to the N-terminus of the protein of interest	[24]
pTEV5	Cloning vector; F1 origin of replication *bla*^*+*^; fuses a TEV-cleavable H_6_ tag to the N-terminus of the protein of interest	[24]
pBAD30	Cloning vector; pACYC184 origin of replication, *bla*^*+*^; expression of the gene of interest under the control of the arabinose-inducible P_araBAD_ promoter	[46]
pBAD18-Kan	Cloning vector; pBR origin of replication, *kan*^*+*^; expression of the gene of interest under the control of the arabinose-inducible P_araBAD_ promoter	[46]

### Site-directed mutagenesis

All site-directed mutations were introduced into targeted genes using the QuikChange® II XL Site-Directed Mutagenesis Kit (Stratagene); all manipulations of plasmids carrying wild-type or mutant alleles of genes of interest were performed in XL10 Gold® Ultracompetent *E*. *coli* cells (Stratagene).

### Polymerase chain reaction (PCR)

Amplification conditions for site-directed mutagenesis were as follows: 95°C for 1 min, followed by 19 cycles of 95°C for 50 s, 60°C for 50 s, 68°C for 6 min 15 s, ending with 68°C for 7 min. All plasmids carrying mutant *S*. *oneidensis prpF* alleles were sequenced using two described primers [19]. DNA sequencing reactions were performed using BigDye***®*** (Applied Biosystems), were purified using CleanSEQ protocols (Agentcourt Biotechnology), and were resolved at the University of Wisconsin-Madison Biotechnology Center.

### Plasmid pPRP195

The *S*. *oneidensis acnD*^*+*^ gene was amplified from plasmid pPRP141 (18) using primers 5’–GTT ATG AGC ACA CAT ATG AAC ACC CAA TAT C– 3’ and 5’ -GAT ATA GGC GGG ATC CAT GTC GGC ATT GC-3’. The resulting DNA fragment (~2.5 kb) was extracted from the gel using the QIAQuick Gel Extraction kit (Qiagen), the fragment was digested with NdeI and BamHI, and ligated into plasmid pET-15b (*bla*^*+*^) digested with the same enzymes; the resulting plasmid (pPRP195) was electroporated into *E*. *coli* DH5α/F’, and cells were plated onto LB *+* ampicillin medium.

### Plasmid pPRP205

Plasmid pPRP195 was digested with NdeI and BamHI; the fragment containing the *S*. *oneidensis acnD*^*+*^ allele was extracted from the gel as described above. The fragment was ligated into plasmid pTEV5 [24] digested with the same enzymes. The resulting plasmid (pPRP205) was electroporated into *E*. *coli* DH5α/F’, and cells were plated onto LB *+* ampicillin medium.

### Plasmids pPRP215-226

Plasmids pPRP153 [18] and pPRP196 [19] were used as templates to generate single-amino acid variants of the *So*PrpF protein with the QuikChange® II XL Site-Directed Mutagenesis kit (Stratagene). Other information pertinent to the construction of these plasmids is summarized in Table 2.

**Table 2:** List of plasmids encoding PrpF variants used in this study.

Plasmid	Allele #	Primers 5’– 3’	Template	Protein Encoded
pPRP215	*prpF255*	GTGGATTGGAGTGGTAACGCGGGTAACTTAACAGCCGCCGGCGGCTGTTAAGTTACCCGCGTTACCACTCCAATCCAC	pPRP153	C107A
pPRP216	*prpF256*	GGATTGGAGTGGTAACAGCGGTAACTTAACAGCCGGCTGTTAAGTTACCGCTGTTACCACTCCAATCC	pPRP153	C107S
pPRP217	*prpF256*	GGATTGGAGTGGTAACAGCGGTAACTTAACAGCCGGCTGTTAAGTTACCGCTGTTACCACTCCAATCC	pPRP196	C107S
pPRP218	*prpF257*	CAACTTCTAGCACCAGCGCGACGGTTATACTGTCACGTGACAGTATAACCGTCGCGCTGGTGCTAGAAGTTG	pPRP153	K73A
pPRP219	*prpF258*	GCAACTTCTAGCACCAGCGAAACGGTTATACTGTCACACGTGTGACAGTATAACCGTTTCGCTGGTGCTAGAAGTTGC	pPRP153	K73E
pPRP220	*prpF257*	CAACTTCTAGCACCAGCGCGACGGTTATACTGTCACGTGACAGTATAACCGTCGCGCTGGTGCTAGAAGTTG	pPRP196	K73A
pPRP221	*prpF258*	GCAACTTCTAGCACCAGCGAAACGGTTATACTGTCACACGTGTGACAGTATAACCGTTTCGCTGGTGCTAGAAGTTGC	pPRP196	K73E
pPRP223	*prpF259*	CAACTTCTAGCACCAGCATGACGGTTATACTGTCACGTGACAGTATAACCGTCATGCTGGTGCTAGAAGTTG	pPRP153	K73M
pPRP225	*prpF255*	GTGGATTGGAGTGGTAACGCGGGTAACTTAACAGCCGCCGGCGGCTGTTAAGTTACCCGCGTTACCACTCCAATCCAC	pPRP196	C107A
pPRP226	*prpF259*	CAACTTCTAGCACCAGCATGACGGTTATACTGTCACGTGACAGTATAACCGTCATGCTGGTGCTAGAAGTTG	pPRP196	K73M

### Isolation of proteins

*So*PrpF protein was purified as described [19]. Variant *So*PrpF proteins were purified using the Maxwell™ 16 system (Promega). Cultures were grown in LB + ampicillin at 37°C, and induced overnight with isopropyl-ß-D-thiogalactopyranoside (IPTG, 0.3 mM) at an OD_650_ of ~0.8. Recombinant PrpD, apo-AcnA and apo-AcnB proteins from *S*. *enterica* were purified from *E*. *coli* as His-tagged proteins as described [15]. *So*AcnD protein was purified as follows. Plasmid pPRP205 (*S*. *oneidensis acnD*^*+*^) was introduced into *E*. *coli* strain BL21 (λDE3) by electroporation selecting for ampicillin resistance on LB agar + ampicillin. Single colonies were used to inoculate 20 ml of LB + ampicillin; cultures were grown overnight at 37°C. Overnight cultures were used to inoculate two liters of LB + ampicillin, and cells were grown at 37°C until the culture reached an OD_650_ of ~0.7. At that point, expression of the plasmid-encoded *S*. *oneidensis acnD*^*+*^ gene was induced by the addition of IPTG (0.3 mM) to the medium, followed by an 18-h incubation period at 37°C. Cells were harvested by centrifugation at 8,000 x *g* at 4°C in 1-liter bottles using a JLA-8.l rotor and a Beckman/Coulter Avanti™ J-20 XPI centrifuge. Cells were broken by sonication (10 min, 50% duty, 5 s pulses, maximal setting) with a 550 Sonic Dismembrator (Fisher Scientific). Cell debris was removed by centrifugation at 39,000 x *g* for 20 min in a Beckman JA 25.5 rotor. The supernatant was filtered through a 0.45 μm filter (Thermo Fisher Scientific). Protein was isolated using Ni-chelate affinity chromatography on Novagen’s His Bind® Resin following the manufacturer’s protocols.

### Crystallization and structural determination of *So*PrpF^K73E^

*So*PrpF^K73E^ was screened for initial crystallization conditions in a 144-condition sparse matrix screen developed in the Rayment laboratory (unpublished information). Single, diffraction quality crystals were grown by hanging drop vapor diffusion by mixing 2 μL of 28 mg/mL *So*PrpF^K73E^ in 2-amino-2-(hydroxymethyl)propane-1,3-diol hydrochloride buffer (Tris-HCl, 10 mM, pH 7.6) containing NaCl (50 mM) with 2 μL well solution containing 2-(*N*-morpholino)ethanesulfonic acid buffer (MES, 100 mM, pH 6.0) containing sodium malonate (136 mM), polyethylene glycol 4000 (PEG 4K, 15% w/v) at room temperature. Hanging droplets were nucleated after 24 h from an earlier spontaneous crystallization event using a cat’s whisker. Crystals grew to approximate dimensions of 200 X 200 X 400 μm within 3 days. The crystals were transferred directly to a cryoprotecting solution that contained MES buffer (100 mM, pH 6.0), sodium malonate (136 mM), PEG 4K (30% w/v) and vitrified by rapid plunging into liquid nitrogen. *So*PrpF^K73E^ crystallized in the space group P2_1_ with unit cell dimensions of *a* = 51.8 Å, *b* = 103.4 Å, *c* = 78.1 Å and two chains in the asymmetric unit.

X-ray diffraction data were collected on a Pilatus detector at SBC Beamline 19-ID (Advanced Photon Source, Argonne National Laboratory, Argonne, IL) The X-ray data were processed and scaled using the HKL-2000 program that integrates data collection, data reduction, phasing and model building [25]. Relevant X-ray diffraction data collection statistics are presented in Table 3. The previously determined model for *So*PrpF apo structure (PDB ID: 2PVZ) was used as the search model to solve the *So*PrpF^K73E^ apo structure via molecular replacement with the program Phaser [26]. Alternate cycles of manual model building and least squares refinement with the programs COOT [27] Refmac [28] and Phenix [29] reduced the R-factor to 16.5% for all X-ray data from 50–1.22 Å. Relevant refinement statistics are presented in Table 3.

**Table 3:** Data collection and refinement statistics.

Data collection	PrpF^K73E^
Space group	P2_1_
Unit-cell	*a* = 51.8
parameters (Å)	*b* = 103.4
	*c* = 78.1
	*ß* = 104.47°
Wavelength	0.979
resolution range (Å)	50–1.22(1.24–1.22)^a^
reflections: measured	2973055
reflections: unique	227930
Redundancy	13.0 (6.1)
Completeness (%)	96.5 (84.5)
average I/	49.5 (2.4)
Rsym ^b^ (%)	5.3 (67.1)
*R*work ^c^ (%)	16.4 (23.2)
*R*free (%)	18.4 (28.6)
no. protein atoms	5907
no. water molecules	1013
Wilson B-value (Å^2^)	14.5
Average B factors (Å^2^)	
PrpF monomer A	17.2 for 2956
PrpF monomer B	19.1 for 2923
Ligand	16.5 for 28
Solvent	29.2 for 1013
Ramachandran (%)	
Most favored	98.0
Additionally	2.0
allowed	
Generously	0.0
allowed	
Disallowed	0.0
rms deviations	
Bond Lengths (Å)	0.006
Bond angles (Å)	1.066
Chiral	

^a^Data in parentheses represent highest resolution shell.

^b^*R*sym = Σ*|*I(hkl)–I*|* / Σ*|*I(hkl)*|*, where the average intensity *I* is taken over all symmetry equivalent measurements and *I*_(hkl)_ is the measured intensity for a given reflection.

^c^*R*factor = Σ*|*F(obs)–F(calc)*|* / Σ*|*F(obs)*|*, where *R*work refers to the *R*factor for the data utilized in the refinement and *R*free refers to the *R*factor for 5% of the data that were excluded from the refinement.

### Reactivation of aconitases

The Fe/S centers of *S*. *enterica* AcnA (hereafter *Se*AcnA), AcnB (hereafter *Se*AcnB), and *So*AcnD were reactivated using described protocols without modifications [30, 31]. All solutions used were freed of dissolved O_2_ by degassing as described [32, 33].

### High-performance liquid chromatography (HPLC)

Enzyme-dependent dehydration of citrate and 2-methylcitrate was performed in 1-ml reaction mixtures containing Tris-HCl buffer (90 mM, pH 8.0 at 25°C), citrate or 2-MC (5 mM), and *So*AcnD (10 μg of reactivated protein) or *S*. *enterica* PrpD (hereafter *Se*PrpD, 13 μg). Reaction mixtures were incubated for 1.5 h at 37°C. Reactions were stopped by the addition of 10 N H_2_SO_4_ to a final concentration of 5 mM. Particulate matter was removed from reaction products by filtration using a Spin-X® centrifuge tube filter (Costar), and products were resolved by HPLC using a Beckman/Coulter chromatograph equipped with an Aminex® HPX-87H HPLC organic acid analysis column (BioRad) equilibrated and developed isocratically with H_2_SO_4_ (5 mM). Elution of compound off the column was detected by monitoring the absorbance at 210 nm.

### Kinetic analysis of *S*. *enterica* aconitases

All reactions were performed in Tris-HCl buffer (50 mM, pH 8.0 at 25°C) containing KCl (100 mM). Enzyme was incubated in buffer for 3–5 min before reactions were initiated by the addition of either *cis-*aconitate or 2-methy-*cis*-aconitate as substrate. Reactions were performed in triplicate, and their progress was monitored using a Perkin Elmer (Norwalk, CT) Lambda 40 UV/Vis Spectrometer at 240 nm; temperature in the cuvettes was maintained with a circulating water bath set at 37°C. Data collection and analysis was performed with Perkin Elmer UV Kinlab software. An extinction coefficient of 3800 M^-1^ cm^-1^ was used for *cis*-aconitate [34], and 4690 M^-1^ cm^-1^ was calculated for the chemically synthesized 2-methyl-*cis*-aconitate. Kinetic curves were each repeated three times, with each substrate concentration tested in triplicate. The reported values are the median of all three experiments. Data did not deviate more than 15% from the median value.

### *In vivo* assessment of activity associated with variant *So*PrpF proteins

Growth curves of *S*. *enterica* strains were performed in NCE medium supplemented with succinate (30 mM, pH 7.0 at 25°C) or propionate (30 mM, pH 7.0 at 25°C) as carbon and energy source. In both cases a low concentration of glycerol (1 mM) was added to accelerate the catabolism of succinate or propionate. Strains were grown overnight in LB medium supplemented with the appropriate antibiotic. Two microliters of an overnight culture were used to inoculate 198 μl of NCE medium in a 96-well microtiter plate. Growth was monitored using an EL808^TM^ microplate reader (Bio-Tek Instruments) with the incubation chamber set at 37°C. Absorbance readings were recorded every 15 min at 630 nm with 850 s of shaking between readings. All cultures were grown in triplicate. Growth curves were plotted using Prism v4.0 software (GraphPad Software).

### *In vitro* assessment of activity associated with variant *So*PrpF proteins

To analyze the activity of *So*PrpF variants, the product of the *So*AcnD enzyme was synthesized as follows. Reactivated *So*AcnD was incubated overnight at 30°C with 2-MC (1 mM) in Tris-HCl buffer (50 mM, pH 8.0) containing KCl (100 mM). *So*AcnD protein was removed from the mixture by the addition of His-Mag^TM^ Agarose Beads (Novagen) resuspended in Tris-HCl buffer (50 mM, pH 8.0) containing KCl (100 mM). His-Mag^TM^ beads were removed, and reaction mixtures were pooled. *So*PrpF protein (100 ng) was added to the reactions, samples were taken as a function of time, and were resolved by HPLC as described above.

## Results

### *So*AcnD and *Se*PrpD synthesize different methylaconitate isomers

We previously showed that *So*PrpF has isomerase activity that can convert *cis*-aconitate to *trans-*aconitate [19]. Given that *So*PrpF isomerase activity is needed to restore growth of a *S*. *enterica prpD* strain with propionate, we surmised that *Se*PrpD and *So*AcnD must synthesize two different methylaconitate isomers, and that *So*PrpF isomerizes the *So*AcnD product into the *Se*PrpD product, so that *Se*AcnB, the next enzyme in the 2-MCC, can synthesize 2-methylisocitrate (Fig 1).

**Fig 2:**
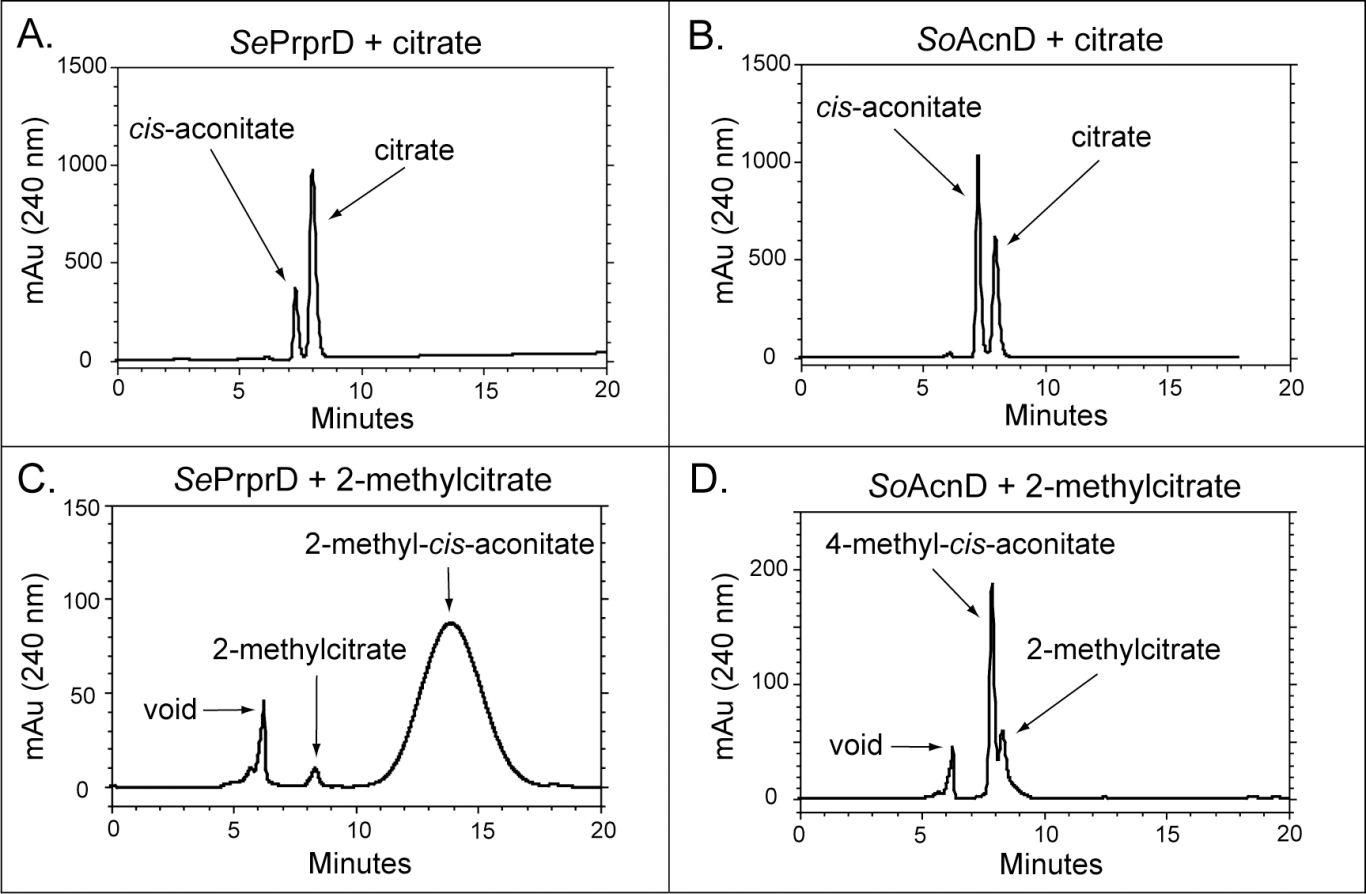
HPLC analysis of PrpD and AcnD reaction products. Reactions products when *Se*PrpD used citrate (panel A) or 2-methylcitrate (panel B) as substrate. Reaction products when *So*AcnD used citrate (panel B) or 2-methylcitrate (panel D) as substrate. Reaction mixtures were resolved by HPLC using a BioRad Aminex® HPX-87H Organic Acids Analysis column developed isocratically with 5 mM H_2_SO_4_ as the mobile phase. Elution was monitored at 240 nm.

To test this hypothesis, we determined whether *Se*PrpD and *So*AcnD proteins synthesized different isomers of aconitate and methylaconitate. To do this, we incubated *Se*PrpD and *So*AcnD with citrate or 2-methylcitrate (each at 5 mM) in 1-ml reaction mixtures. After a 1.5-hr incubation period, the reactions mixtures were acidified with H_2_SO_4_ to a final concentration of 5 mM, and a 100 μl sample of the reaction was resolved by HPLC. When incubated with citrate, *Se*PrpD (Fig 2A) and *So*AcnD (Fig 2B) converted citrate (retention time ~8 min) into *cis*-aconitate (retention time ~7.2 min). When incubated with 2-methylcitrate, *Se*PrpD synthesized a product that eluted off the column as a broad peak centered at ~13.8 min (Fig 2C). On the other hand, *So*AcnD synthesized a product that eluted as a sharp peak at ~8 min (Fig 2D). The different retention times and chromatographic behavior indicated to us that the dehydration product of *Se*PrpD and *So*AcnD were different compounds. Analysis of chemically synthesized 2-methyl-*cis*-aconitate revealed a peak that matched the product of the *Se*PrpD reaction (Fig 2C), indicating that *Se*PrpD synthesized 2-methyl-*cis*-aconitate from 2-methylcitrate.

### 2-Methyl-*cis*-aconitate is a substrate of *Se*AcnA and *Se*AcnB

The HPLC chromatograms of the products of the *Se*PrpD and *So*AcnD reactions suggested that the *Se*PrpD product, 2-methyl-*cis*-aconitate, was the substrate that aconitases rehydrated to yield 2-methylisocitrate agreeing with previously reported data [35, 36]. Since *So*AcnD cannot support growth with propionate in the absence of *So*PrpF, we surmised that the product of *So*AcnD was not a substrate of aconitases, but the product of the *So*PrpF reaction was.

Kinetic analysis of the *S*. *enterica* aconitases indicated that both, *Se*AcnA and *Se*AcnB, used 2-methyl-*cis*-aconitate as substrate to yield 2-methylisocitrate, albeit at a significantly slower rate than *cis*-aconitate, the TCA cycle intermediate (Table 4). The catalytic efficiencies of *Se*AcnA and *Se*AcnB were 40 and 60 lower when 2-methyl-*cis*-aconitate was the substrate than when *cis*-aconitate was the substrate, respectively. When 2-methyl-*cis-*aconitate ws the substrate, *K*_*m*_ values ranged from 180 to 229 μM and *V*_*max*_ values ranged from 50 to 60 μM min^-1^. The numbers reported here are representative of three kinetic studies.

**Table 4:** Kinetics of AcnA and AcnB with *cis*-aconitate and 2-methyl-*cis*-aconitate.

Isomer/Enzyme used	*K*_*m*_ (μM)	*V*_*max*_ (μM min^-1^)	*k*_*cat*_ (min^-1^)	*k*_*cat*_ / *K*_*m*_ (min^-1^ μM^-1^)
***cis*-aconitate**				
AcnA	22 ± 2	86 ± 2	3739 ± 87	170
AcnB	17 ± 3	21 ± 1	2091 ± 100	123
**2-methyl-*cis*-aconitate**				
AcnA	210 ± 35	50 ± 3	708 ± 42	3.4
AcnB	208 ± 39	10 ± 1	396 ± 40	1.9

All kinetic reactions were performed in triplicate and individual kinetic curves were also repeated in triplicate. Reported values represent median values from all three experiments. No value differed more than 15% from the median value.

### *So*PrpF isomerizes the *So*AcnD reaction product to 2-methyl-*cis*-aconitate

**Fig 3:**
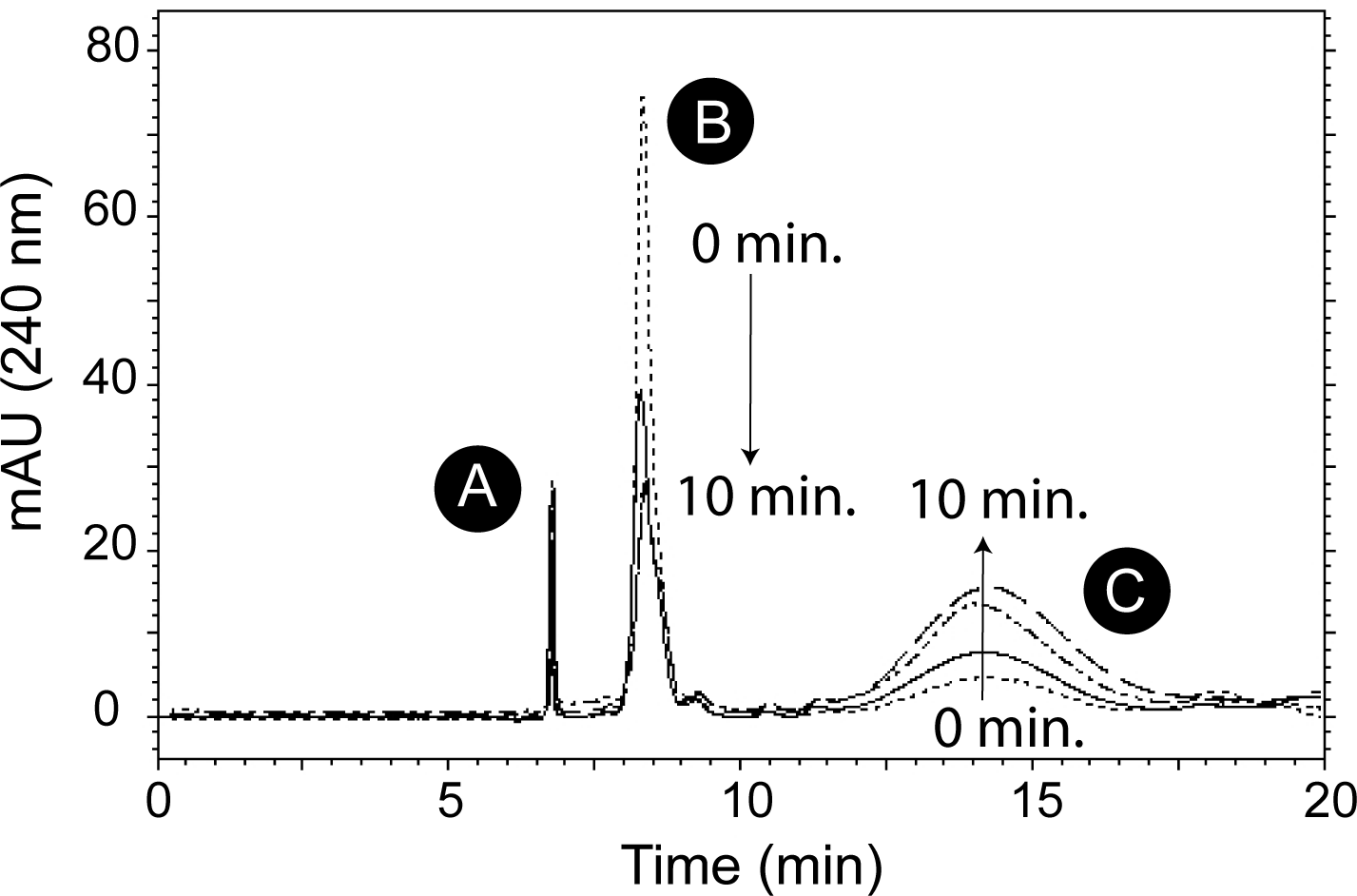
*So*PrpF isomerizes the *So*AcnD product into 2-methyl-*cis*-aconitate. Reactivated *So*AcnD was incubated with 2-MC, once *So*AcnD was removed from the reaction, PrpF was added (100 μg) and samples were removed at the indicated times stopped by the addition of H_2_SO_4_ to 5 mM. Reactions were resolved by HPLC using an Aminex HPX-87H Organic Acid column equilibrated and isocratically developed with 5 mM H_2_SO_4_; reaction products were monitored at 240 nm. A. void; B. 4-methyl-*cis-*aconitate C. 2-methyl-*cis-*aconitate.

We tested whether *Se*AcnA and *Se*AcnB used the *So*AcnD reaction product as substrate (data not shown). The latter was synthesized from 2-MC using *So*AcnD. After the *So*AcnD enzyme was removed from the reaction mixture, either *Se*AcnA or *Se*AcnB was added, and the change in absorbance at 240 nm was monitored. Neither *Se*AcnA nor *Se*AcnB used the *So*AcnD reaction product as substrate. Purified *So*PrpF protein (100 μg) was added to the reaction mixture, and the reaction was allowed to proceed for one hour at 30°C. Addition of *Se*AcnA resulted in specific activities of approximately 14 ± 1 nmol min^-1^ μg^-1^ of protein, while addition of *Se*AcnB resulted in specific activities of 48 ± 4 nmol min^-1^ μg^-1^ of protein. Additionally, HPLC analysis of the products of the *So*PrpF reactions indicated that *So*PrpF converted the *So*AcnD reaction product into 2-methyl-*cis*-aconitate over time (Fig 3), which can then be used as a substrate by the aconitase enzymes.

### Analysis of variants to gain insights into the mechanism of *So*PrpF catalysis

**Fig 4:**
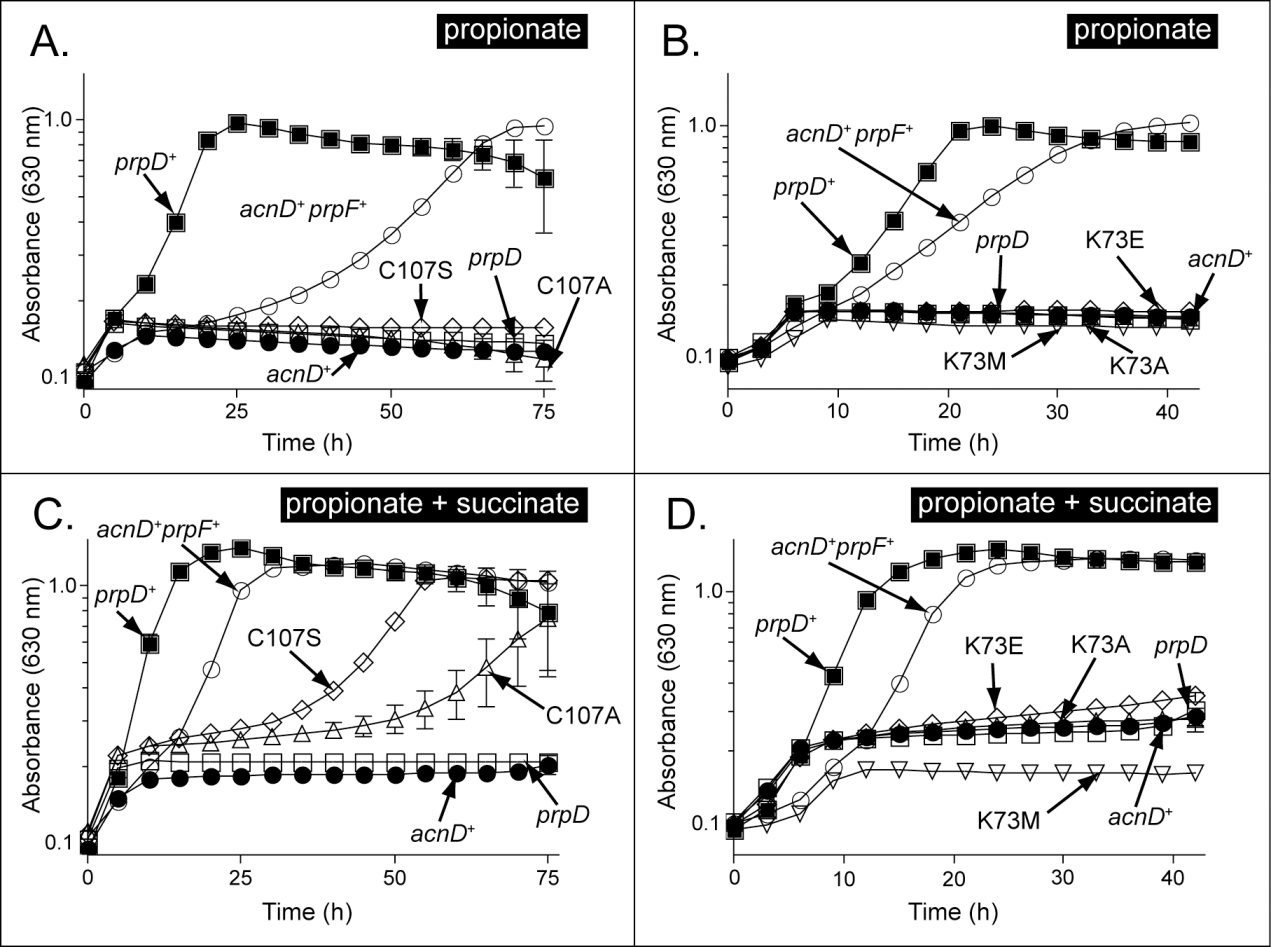
Growth behavior analysis of strains synthesizing variants of PrpF. Growth curves were performed in NCE minimal medium supplemented with propionate (30 mM) (Panels A, B) or propionate + succinate (30 mM ea.) (Panels C, D). Plasmids encoding PrpF variants with substitutions at position C107 failed to restore growth of strains JE9373 (*prpD /* pAcnD^WT^ pPrpF^C107A^) or JE9374 (*prpD* pAcnD^WT^ pPrpF^C107S^) with propionate (Panel A: PrpF^C107S^, open diamonds; PrpF^C107A^, open triangles, respectively), but did restore growth with propionate + succinate, albeit at a slower rate (panel C, diamonds, triangles, respectively). In contrast, substitutions at position K73 failed to compensate for the absence of PrpD on either propionate (panel B: PrpF^K73A^, open triangles; PrpF^K73E^, open diamonds, PrpF^K73M^, open inverted triangles), or succinate + propionate (Panel D: PrpF^K73A^, open triangles; PrpF^K73E^, open diamonds; PrpF^K73M^, open inverted triangles).

Previous crystallographic data identified residues C107 and K73 in the active site of *So*PrpF as likely to be involved in catalysis. Residue C107 is formally equivalent to the catalytic base of diaminopimelate epimerase [19, 37, 38], and residue K73 is in a structurally equivalent position to the catalytic glutamate of the phenazine biosynthetic protein PhzF from *Pseudomonas fluorescens* [39]. We investigated the involvement of these two residues in *So*PrpF catalysis, using site-directed mutagenesis to introduce amino acids changes. Five *So*PrpF variants were constructed. Residue C107 was changed to either Ala or Ser, and residue K3 was changed to Ala, Glu, or Met. Plasmid pPRP153 (*prpF* in pBAD18-Kan) was used in *in vivo* complementation studies using a *S*. *enterica prpD* strain to assess the effect of specific substitutions on PrpF function. Substitutions at C107 (Fig 4A) and at K73 (Fig 4B) resulted in proteins that failed to support growth with propionate as a sole carbon and energy source in a *S*. *enterica* Δ*prpD* strain harboring a plasmid expressing *S*. *oneidensis acnD*^*+*^. However, when growing on medium containing succinate and propionate (30 mM each), a much less stringent test for propionate utilization, both proteins with substitutions at C107 supported propionate catabolism (Fig 4C), albeit at a slower growth rate than the wild-type *prpF* allele, suggesting the presence of proteins with lower activity that could have been caused by misfolding. In contrast, none of the variants with substitutions at residue K73 supported growth under either condition (Fig 4D) suggestive of proteins that either lacked catalytic activity or were misfolded. We note that when grown on medium containing both succinate and propionate, the final cell density of the cultures was approximately 1.3–1.5 A_630_ units, which was substantially higher than the final density of approximately 1.0 A_630_ unit when growing with propionate alone, suggesting that the strains containing the mutant alleles encoding *So*PrpF variants used propionate and succinate as carbon and energy sources.

To assess the residual level of isomerase activity associated with variant *So*PrpF variants, appropriate mutations were introduced into plasmid pPRP196 (*prpF*^+^ in pTEV4); proteins containing the C107A or K73A substitutions were not overproduced and could not be purified. The remaining variants were isolated, and their activity was quantified. None of the variants had any detectable activity. While this may be expected for substitutions of K73, the proposed catalytic residue, it is unclear why the C107S mutant had no activity, especially since the variants were active *in vivo*. More studies may need to be undertaken to fully understand this result.

### Analysis of the tertiary and quaternary structures of the catalytically inactive *So*PrpF^K73E^ variant

To further understand the roles of residues K73 and C107, we solved the three-dimensional crystal structure of Apo-*So*PrpF^K73E^, which crystallized in space group P2_1_ with cell dimensions *a =* 51.8 *b =* 103.4 *c =* 78.1 Å, and contained two monomers per asymmetric unit. The structure was determined at 1.22Å resolution by molecular replacement using apo-*So*PrpF (PDB ID: 2PVZ) as a search model (Table 3). The two monomers in the asymmetric unit are related by a non-crystallographic twofold axis. The two monomers are highly similar where the rms difference between 381 α-carbon atoms is 0.13Å. Given the similarity between the two monomers, all of the discussion of the structure of a single protein chain is based on that of subunit A.

**Fig 5:**
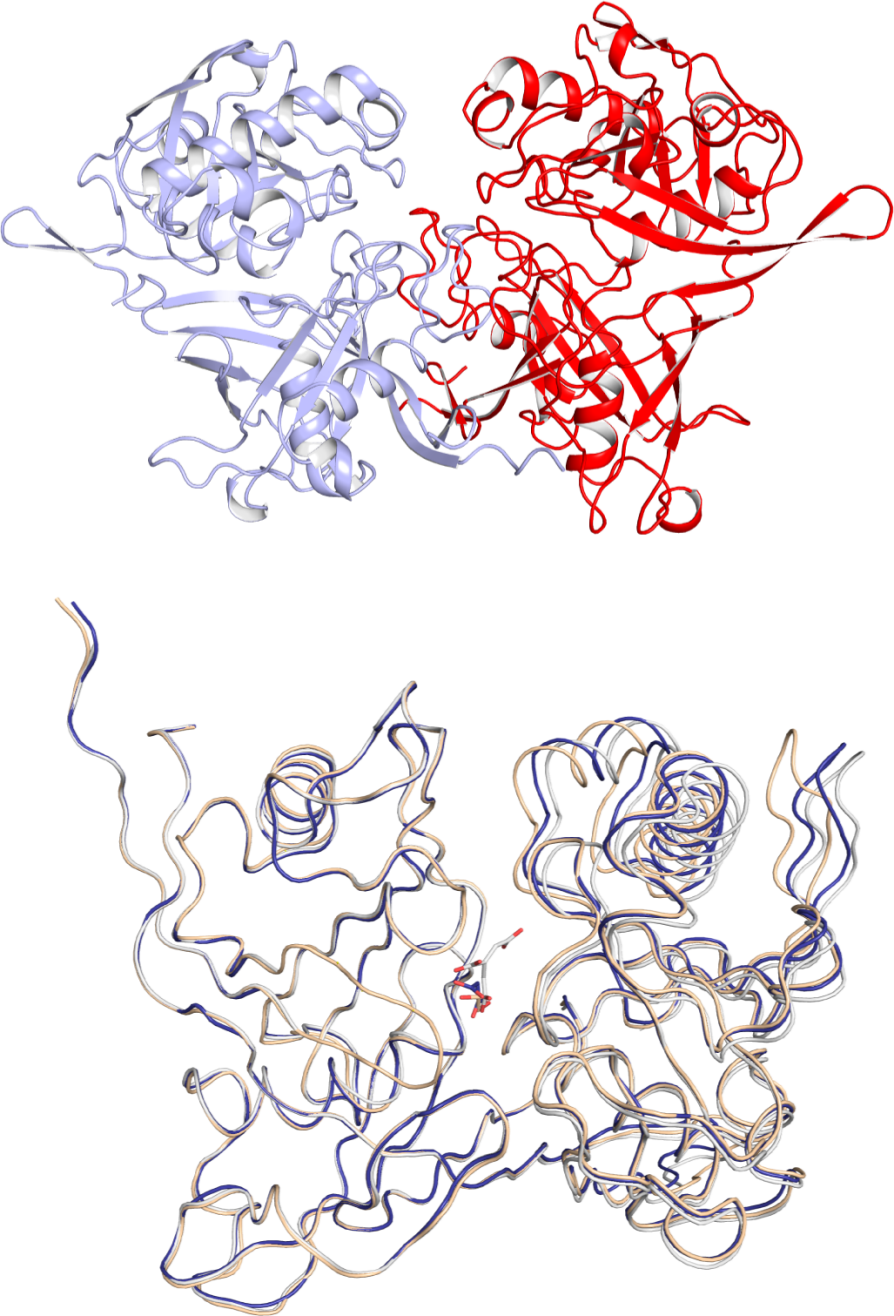
Crystal structure of *So*PrpF^K73E^ at 2.35Å resolution. **A.** Ribbon representation of the *So*PrpF^K73E^ homodimer (*light blue* = monomer A, *red* = monomer B). **B.** Overlay of *So*PrpF^K73E^ (*blue)*, *So*PrpF in complex with *trans*-aconitate (*white*, RCSB accession number 2PW0) and apo-*So*PrpF^WT^ (*tan*, RCSB accession number 2PVZ). The superposition was performed by aligning residues 1–185 with the program Superpose [40]. Figs 5 and 6 were prepared with the program Pymol; DeLano Scientific LLC, Palo Alto, CA.

*So*PrpF assembles to form a homodimer and buries 2400Å^2^ of surface area per monomer, which represents 15% of each monomer’s total surface area (Fig 5A). While the *So*PrpF^K73E^ variant structure can be superposed closely with both the apo-PrpF^W^T and *trans*-aconitate bound structures with rms differences of 0.6 Å and 0.4 Å over 385 α-carbon respectively, an alignment of the N-terminal domains from residues 5–185 reveals that the C-terminal domain of the *So*PrpF^K73E^ structure rotates into a more open conformation. The C-terminal domain rotates ~4.4° between the extremes provided by the apo (closed) and aconitate (open) bound forms. This rotation occurs around the segments that connect the N-terminal and C-terminal domain (E180-N183 and I379-M380) although there are negligible conformational changes associated with these residues since they represent the fulcrum point for the rotation. As such these residues cannot be viewed as a flexible hinge.

**Fig 6:**
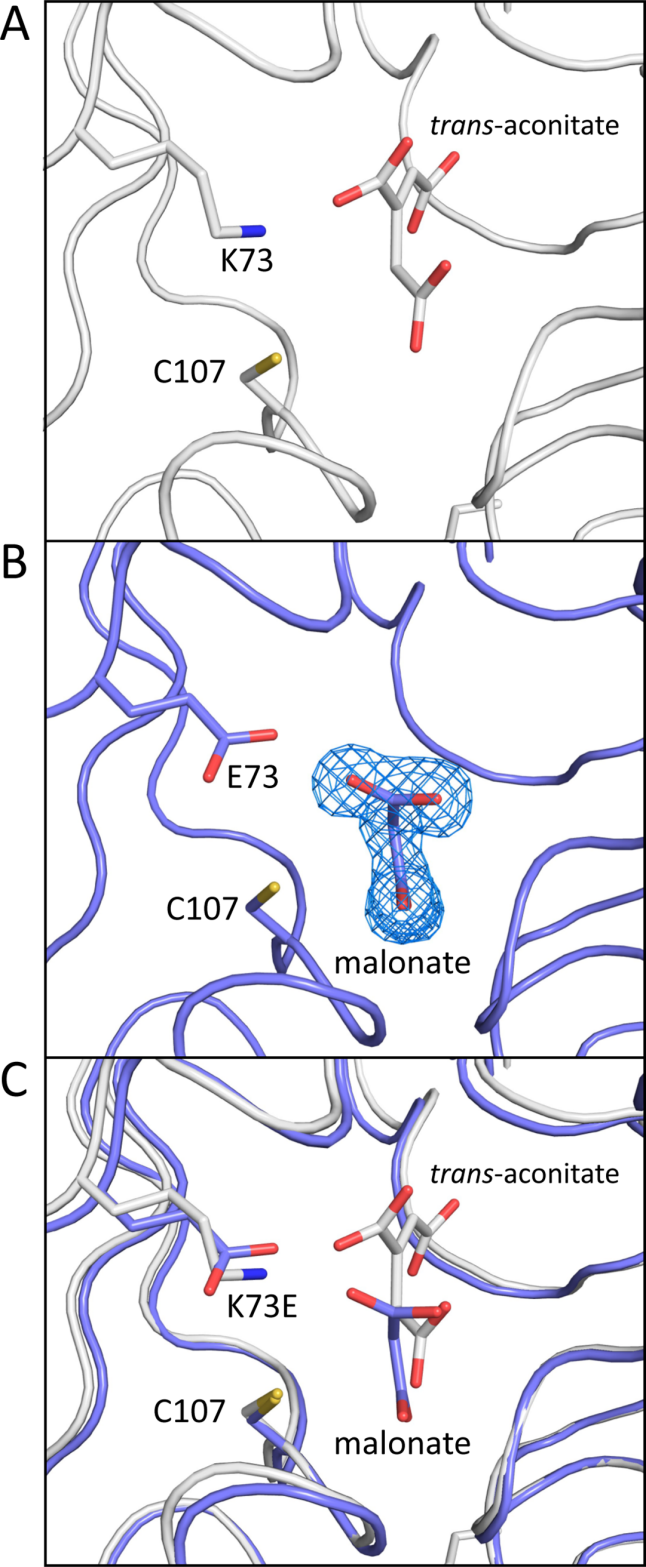
Active site comparisons of *So*PrpF^WT^ and *So*PrpF^K73E^. **A.** Detailed view of the catalytic active site residues, which contact *trans*-aconitate in the *So*PrpF^WT^ protein determined in the presence of *trans*-aconitate (RCSB accession number 2pw0). **B.** Detailed view of the residues lining the active site in the *So*PrpF^K73E^ variant with malonate bound. The electron density map was calculated with coefficients of the form *F*_*o*_*−F*_*c*_ where the ligand was omitted from the final phase calculation refinement and contoured to 4.5 σ. **C.** The superposition of active site residues with *trans*-aconitate and malonate included for reference.

A comparison of the three structures showed that the *trans-*aconitate bound structure was in the most open conformation, while the *So*PrpF^K73E^ structure was intermediate and the apo-*So*PrpF^WT^ structure was in the most closed conformation (Fig 5B). This was not surprising as a glycerol molecule, derived from the cryoprotectant used in the structural determination, was bound in the active site of the apo-*So*PrpF^WT^ structure. Glycerol is a smaller ligand than the true substrate and thus allows the C-terminal domain to move closer to the N-terminal domain since it is not impeded by the larger *trans*-aconitate molecule. Similarly, a malonate molecule derived from the crystallization solution was bound in the active site of the *So*PrpF^K73E^ structure. Because malonate is larger than glycerol and smaller than *trans*-aconitate, the C-terminal domain adopted an intermediary conformation. Thus, the structure of the apo-*So*PrpF^K73E^ variant protein provided an estimate of the conformational freedom available to the N- and C-terminal domains of PrpF. Attempts to obtain substrate complexed *So*PrpF^K73E^ crystals by co-crystallization or soaking into apo crystals with either *trans*-aconitate or 2-methylcitrate were not successful. This difficulty in co-crystallization was not surprising since binding of *trans*-aconitate into the *So*PrpF^K73E^ active site would position a substrate carboxyl moiety within 3 Å of the new glutamate carboxyl group of *So*PrpF^K73E^ variant, thus creating an unfavorable interaction (Fig 6).

### Phylogenetic analysis of PrpF homologues

**Fig 7:**
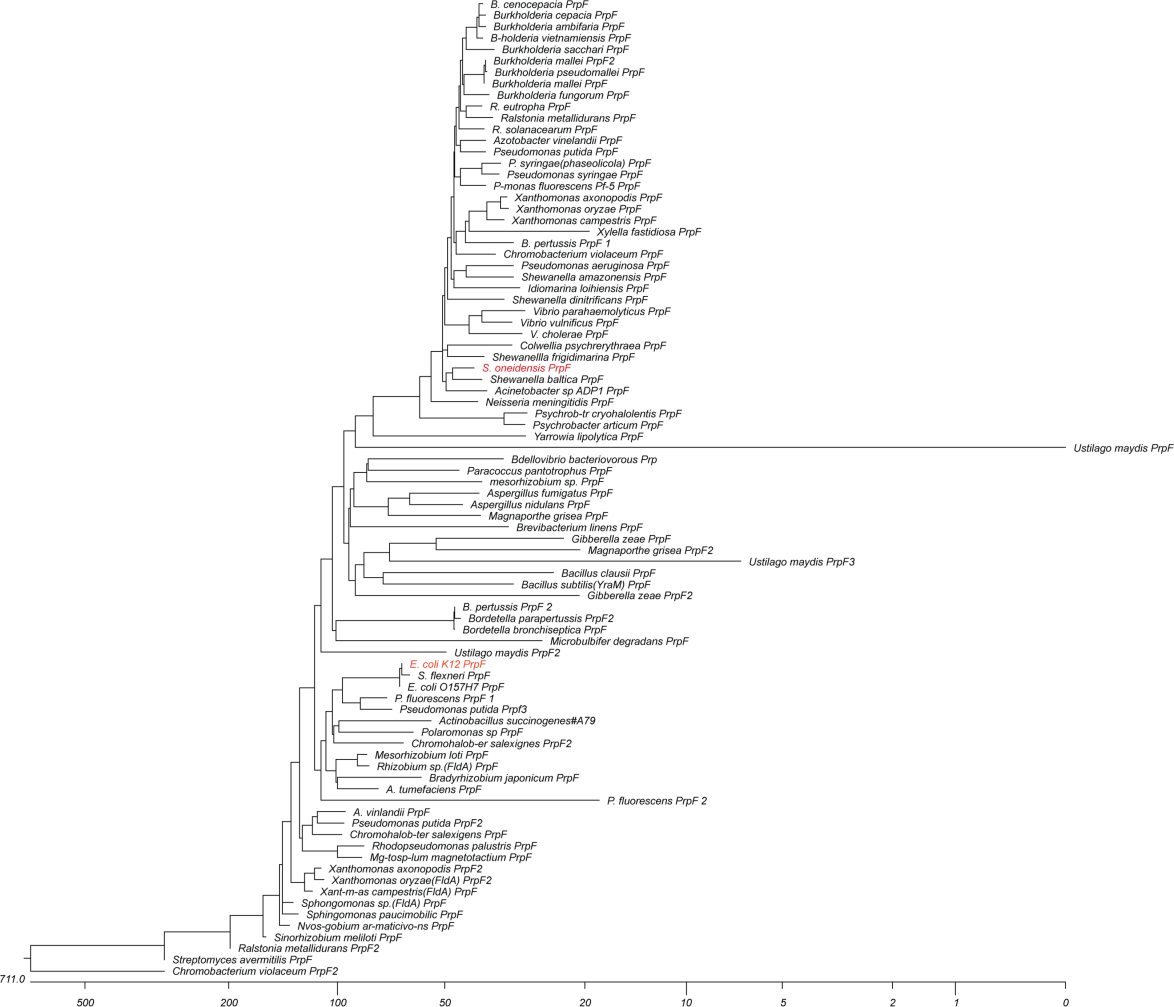
Phylogenetic tree of selected PrpF homologues in nature. Phylogenetic tree of selected PrpF homologues. Eighty-six sequences were selected and aligned using ClustalW software. Proteins towards the top of the tree are mostly found in operons with genes related to propionate catabolism. Proteins that are more divergent are more likely to be found in alternate genetic contexts. The PrpF protein of *Shewanella oneidensis* and the *E*. *coli* K12 PrpF homologue are highlighted in red. Distance is shown in substitutions per 100 residues.

To provide some perspective of the wide distribution of PrpF homologues in nature, we performed a limited phylogenetic analysis of 70 microbes containing a total of 86 PrpF homologues using the MUltiple Sequence Comparison by Log-Expectation (MUSCLE) software. For this purpose, one hundred sequences were selected and aligned (Fig 7). Proteins displaying the shortest distances from the root (clusters at the top and bottom of the tree, respectively) were mostly found in operons with genes related to propionate catabolism. Proteins that are more divergent were more likely to be found in alternate genetic contexts. In Fig 7, proteins from Gram-positive species are indicated with squares and proteins from fungi are indicated with circles. When an organism possesses multiple copies of the gene, the additional copies that are not found to be associated with propionate catabolic genes are marked with triangles. As can be noted in the tree, proteins found in organisms that are more distant evolutionarily from the Gamma-proteobacteria, and proteins not involved in propionate catabolism tend to be more distant in the phylogenetic tree as well.

## Discussion

In this paper, we show that the PrpF protein of *S*. *oneidensis* (*So*PrpF) has isomerase activity that converts the product of the *So*AcnD reaction into 2-methyl*-cis-*aconitate, which can then be converted into 2-methylisocitrate by aconitase. The *So*AcnD product is likely 4-methyl-*cis*-aconitate. On the basis of the analysis of the crystal structures of *So*PrpF^WT^ and *So*PrpF^K73E^, we propose that *So*AcnD dehydrates 2-methylcitrate into 4-methyl-*cis*-aconitate. We established the order of the 2-MCC in *Shewanella oneidensis* and, by inference, in other bacteria that contain *acnD* and *prpF* homologues in their *prp* operons. That is, in organisms that use the AcnD/PrpF enzymes instead of PrpD, 2-methylcitrate is dehydrated by AcnD to 4-methyl-*cis*-aconitate, which is then isomerized to 2-methyl-*cis-*aconitate by PrpF, and rehydrated to 2-methylisocitrate by aconitases (Fig 1).

### Rationale for the use of the AcnD/PrpF system

It is unclear why some bacteria such as *S*. *oneidensis* and *V*. *cholera* use AcnD and PrpF to synthesize 2-methyl-*cis*-aconitate, while others use a PrpD homologue. This work does not address this interesting question, but it does provide experimental data to support the assignment of a biochemical activity for the *So*PrpF enzyme.

We note that a previous report suggested that the formation of 2-methyl-*cis*-aconitate must occur via a unique *syn* elimination of water [3]. To date, a detailed analysis of the mechanism of *So*AcnD or any of its homologues has not been reported. Thus, for the sake of this discussion, we will assume that the *So*AcnD protein, like most aconitases, is limited to *anti* β-eliminations [41]. Therefore, like other aconitases, *So*AcnD would remove the *pro-R* proton from the oxaloacetate-derived carbon of citrate (C-4), not from the carbon derived from acetate (C-2) [42]. In 2-methylcitrate, carbons 3, 4, and 5 are the carbons derived from oxaloacetate, and as such, carbons 3 and 4 should be where *So*AcnD dehydrates the substrate. The C2 of 2-methylcitrate is derived from propionate, and should not participate in the *So*AcnD-catalyzed dehydration reaction. An *anti* ß-elimination of the *pro-R* proton at C4 should result specifically in the formation of 4-methyl-*cis*-aconitate by *Se*AcnD.

Since *So*AcnD is likely to yield 4-methyl-*cis*-aconitate, the presence of an isomerase, like *So*PrpF, becomes necessary, as the accumulation of 4-methyl-*cis*-aconitate could inhibit aconitase. Isomerization of 4-methyl-*cis*-aconitate to 2-methyl-*cis*-aconitate then allows aconitase to convert the latter to 2-methylisocitrate, which is cleaved by the 2-methylisocitrate lyase yielding succinate and pyruvate (Fig 1). The greater activity of *Se*AcnB on enzymatically-derived 2-methyl-*cis*-aconitate when compared to *Se*AcnA suggests AcnB is the primary aconitase utilized in propionate catabolism, which is in agreement with our previously reported data that indicated *Se*AcnB was the primary aconitase involved in propionate catabolism [15].

### Modifications to the 2-methylcitric acid cycle

The identification of *So*PrpF as a 4-methyl-*cis*-aconitate isomerase resolves the issue of why *So*AcnD and *So*PrpF proteins are required to restore propionate catabolism in a Δ*prpD* strain in *S*. *enterica* (18). Fig 1 reflects the findings of this work, in that it shows the sequence of reactions catalyzed by AcnD and PrpF.

### Why AcnD, and not any of the other aconitases?

It is unclear why any organism would dedicate an aconitase to the 2-MCC, given that several aconitases already exist in the cell. Studies of the conformation of mitochondrial aconitases suggest that the methyl group of 2-methylcitrate may sterically interfere with this compound entering the active site correctly [43]. We suggest that the active site of *So*AcnD may be different enough to use 2-methylcitrate as a substrate.

### Structure-function analysis of the *So*PrpF active site

In our previous work we suggested that active site of PrpF utilized a single catalytic lysine (Lys 73) to catalyze a proton extraction and a conformational rearrangement around the C2-C3 bond [19]. Data presented here indicates that the reaction instead involves an allylic rearrangement and bond migration mechanism of an aconitate isomerase from *Pseudomonas putida* described by Klinman and Rose that may have been a PrpF homologue [44]. However, using an allylic rearrangement mechanism would require two catalytic residues and PrpF appears to only have one. The most likely candidate for a second catalytic residue would be C107, which is formally equivalent to the catalytic residue of diaminopimelate epimerase. A recent publication suggested that 4-methyl-*cis*-aconitate would bind in a manner that would allow the C107 to function as a second catalytic residue [45]. Our mutational analysis sheds light on this possibility. If C107 were required for function, complete loss of enzyme activity in variants with substitutions in C107 would be expected. In Fig 4A, we present *in vivo* evidence in support for a critical role for C107. When propionate was used as the sole source of carbon and energy, the *So*PrpF^C107S/A^ variants failed to support growth of a Δ*prpD* strain expressing *So*AcnD. Although *So*PrpF^C107S/A^ variants retain activity (Fig 4C), it is insufficient to support growth with propionate as a carbon and energy source. Further work is needed to better understand the catalytic mechanism of *So*PrpF.

### Significance of the broad distribution of PrpF homologues

The widespread distribution of genes encoding PrpF homologues in prokaryotes and eukaryotes, reveals the importance of isomerization in cell physiology. While the majority of the bacterial homologues appear to be part of operons along with other propionate utilization genes, many of them are present in operons encoding genes of unknown function. These *prpF* homologues suggest the possibility of double-bond isomerization being involved in many metabolic pathways.

In summary, we report data in support the following conclusions: i) PrpF isomerizes 4-methyl-*cis*-aconitate into 2-methyl-*cis*-aconitate; ii) the product of the AcnD reaction with 2-methylcitrate is most likely 4-methyl-*cis*-aconitate; iii) the aconitase proteins of *S*. *enterica* (*i*.*e*., AcnA, AcnB) only rehydrate 2-methyl-*cis-*aconitate to 2-methylisocitrate; and iv) the proposed catalytic lysine (Lys 73) is absolutely required for activity of PrpF, and while substitutions of C107 do not abolish enzyme activity, PrpF^C107^ variants do not support growth with propionate.

## Conclusions

Work reported in this paper advances our understanding of the 2-methylcitric acid cycle responsible for the conversion of the short-chain fatty acid propionate to pyruvate. The best-characterized sequence of reactions of the pathway involves the PrpD enzyme, which dehydrates 2-methylcitrate to 2-methyl-*cis-*aconitate. However, some microorganisms have replaced PrpD with two proteins, namely AcnD and PrpF, whose functions are not well understood. *In vivo* and *in vitro* evidence presented in this paper support to the conclusion that in an AcnD/PprF-dependent 2-methylcitric acid cycle, AcnD likely generates 4-methyl-*cis*-aconitate, which is isomerized by PrpF into 2-methyl-*cis*-aconitate. Additional work is needed to confirm the identity of the *So*AcnD product. Structural analysis of an inactive variant of PrpF provides insights into its mechanism of function. A better understanding of PrpF activity will be of value to other investigators researching the function of PrpF-like isomerases, which are widely distributed among prokaryotes.

## Supporting information

S1 Fig Kinetics of aconitase product formation. Reaction mixtures contained aconitase A (AcnA) and aconitase B (AcnB) with *cis*-aconitate (Panel A: AcnA; Panel B: AcnB) or 2-methyl-*cis*-aconitate (Panel C: AcnA; Panel D: AcnB) as substrate. Product formation (either isocitrate from *cis-*aconitate; 2-methylisocitrate from 2-methy-*cis-*aconitate) was monitored as a decrease in absorbance at 240 nm over time (min; *y*-axes) as a function of substrate concentration (µM; *x* axes). The increase in the rate at which A_240_ decreased is what is plotted. Detailed assay conditions are described under *Materials and methods*.
(PDF)
