# Supplementary material for: The PrpF protein of Shewanella oneidensis MR-1 catalyzes the isomerization of 2-methyl-cis-aconitate during the catabolism of propionate via the AcnD-dependent 2-methylcitric acid cycle
Source: PLoS One. 2017 Nov 16;12(11):e0188130. doi: 10.1371/journal.pone.0188130 (PMC5690661; doi:10.1371/journal.pone.0188130)

The PrpF protein of *Shewanella oneidensis* MR-1 catalyzes the isomerization of 2-methyl-*cis*-aconitate during the catabolism of propionate via the AcnD-dependent 2-methylcitric acid cycle

Christopher J. Rocco[1,#a], Karl M. Wetterhorn[2], Graeme S. Garvey[2,#b], Ivan Rayment[2], and Jorge C. Escalante-Semerena[5*]

[1]Department of Bacteriology[1], University of Wisconsin, Madison, WI 53706 USA
[2]Department of Biochemistry, University of Wisconsin, Madison, WI 53706 USA
3Department of Microbiology, University of Georgia, Athens, GA 30602 USA
[#a]Present Address: Center for Microbial Pathogenesis, The Research Institute at Nationwide Children's Hospital, Columbus, OH 43210
[#b]Present address: Monsanto Vegetable Seeds, Woodland, CA 95695

Running title: PrpF is a 2-methyl-*cis*-aconitate isomerase

*Corresponding author E-mail: jcescala@uga.edu

Figure S1. Kinetics of aconitase product formation. Reaction mixtures contained aconitase A (AcnA) and aconitase B (AcnB) with *cis*-aconitate (Panel A: AcnA; Panel B: AcnB) or 2-methyl-*cis*-aconitate (Panel C: AcnA; Panel D: AcnB) as substrate. Product formation (either isocitrate from *cis*-aconitate; 2-methylisocitrate from 2-methy-*cis*-aconitate) was monitored as a decrease in absorbance at 240 nm over time (min; *y*-axes) as a function of substrate concentration (µM; *x* axes). The increase in the rate at which A_{240} decreased is what is plotted. Detailed assay conditions are described under Materials and Methods.

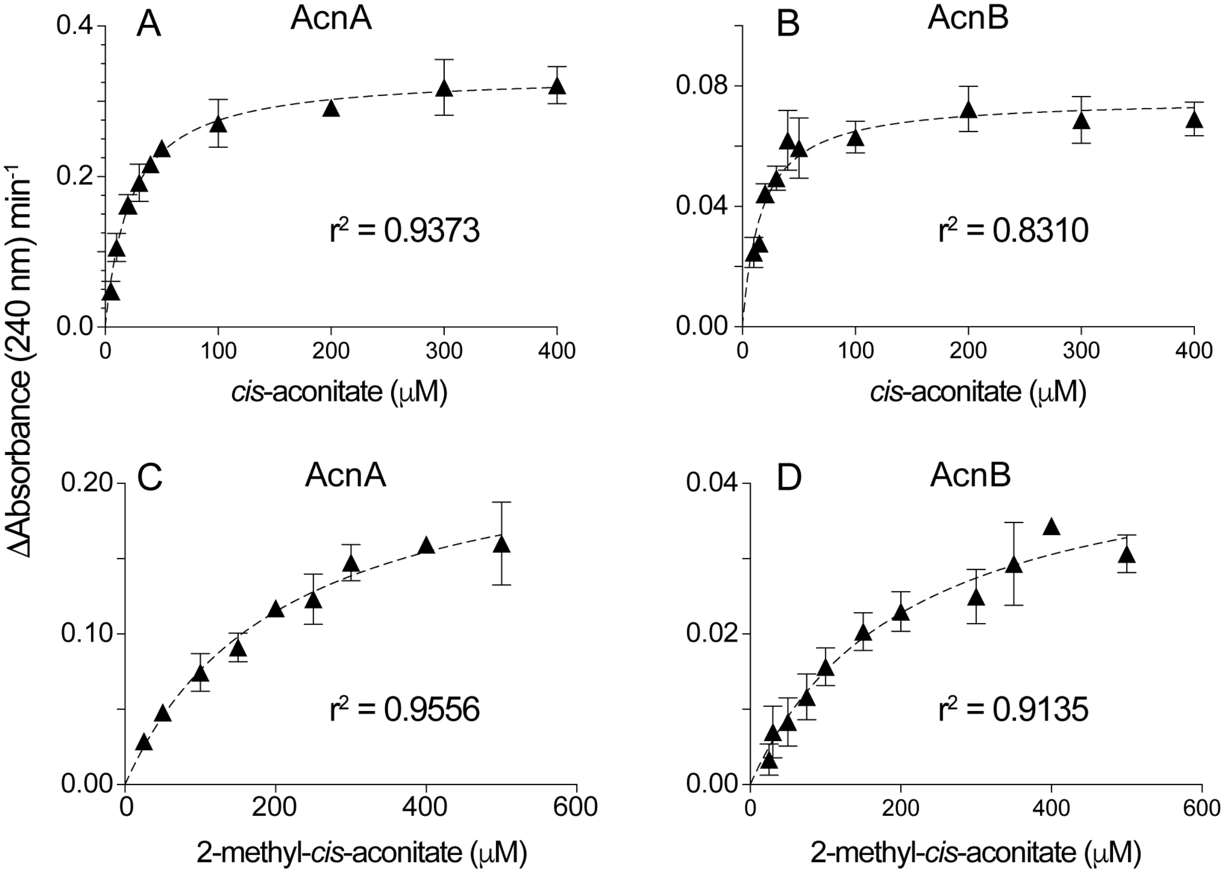

Supplement: S1 Fig — Reaction mixtures contained aconitase A (AcnA) and aconitase B (AcnB) with cis-aconitate (Panel A: AcnA; Panel B: AcnB) or 2-methyl-cis-aconitate (Panel C: AcnA; Panel D: AcnB) as substrate. Product formation (either isocitrate from cis-aconitate; 2-methylisocitrate from 2-methy-cis-aconitate) was monitored as a decrease in absorbance at 240 nm over time (min; y-axes) as a function of substrate concentration (µM; x axes). The increase in the rate at which A240 decreased is what is plotted. Detailed assay conditions are described under Materials and methods. (PDF) [file pone.0188130.s001.pdf]
